# Treatment of Hare Lip

**Published:** 1863-05

**Authors:** David P. Smith

**Affiliations:** Surg. Vols., Surgeon in Charge of Fairfax Seminary Hospital


REMARKS ON THE TREATMENT OF HARE-LIP. *By David P. Smith, Surg. Vols., Surgeon in Charge of Fairfax Seminary Hospital.*—There is perhaps no operation in which accuracy of adjustment is more required than in the operation of hare-lip. Upon the mechanical nicety of the paring of the edges, and the introduction of the pins or sutures, depends the personal appearance of the patient for life. In the case of a fine talented young man, upon whom I recently operated for the cleft palate, the hare-lip accompanying the last named deformity was made, by the mal-adroitness of the surgeons, to require three operations in early life before decent adjustment was effected. I saw a year or two ago a young gentleman of first rank in New York, who consulted me on account of the great deformity which remained after a similar operation which had been performed in early infancy. A child aged five years was recently brought to me with great deformity remaining after the operation for hare-lip. When we consider the very bad effect that the consciousness of deformity has upon the mind of a sensitive child, it may perhaps be thought worth our while to adopt some method that will *insure* good results. Such I believe to be the one which I will endeavor to explain in as brief a manner as possible.

I conceive that

1st. The hæmorrhage should be reduced to a minimum.

2d. The paring of the margins should be done in a manner that will prevent any notch in the free margin, and will also enable the incision to be exact in spite of the struggles of the infant.

3d. Insertion of the needles or sutures should be rendered mathematically exact.

4th. The time occupied should be reduced to a minimum.

The experience of the profession has furnished principles that will be sufficient to enable us to carry out the above indications, if we will but seek. Take two of the forceps invented a few years ago by Dr. Alden March of Albany; provide them with catches like artery forceps, and have transverse lines marked upon them at distances of a quarter of an inch, so that a means may be furnished of exactly and rapidly determining the situation of the sutures. I modify the *method* of operating by applying these two exactly similar forceps, one to each side of the cleft, fastening them there by the catch introducing the sutures about half an inch from the margin of the forceps, exactly opposite the transverse lines, and finally cutting away the margins of the cleft inclosed in the forceps. Nothing now remains to be done but to close the cleft, which, on account of the mechanically exact introduction of the sutures, is effected instantaneously. Metallic sutures being used their prior introduction does not expose them to the danger of being cut. According to Lisfranc's method I would not at the labial margin entirely sever the portion pared from the margin of the cleft, but would suffer it to remain on each side, until the cleft being accurately adjusted these *parings* may be shortened to suit, a suture being passed through them, forming a decided V-shaped projection downwards. I deem this precaution necessary in addition to curving the lines of incision after the manner of Celsus, because I never yet saw any one years after the operation in whom an umseemly no:ch did not exist.

I forward to you these few remarks concerning this common operation, written two or three years ago, because recently a soldier was admitted into this hospital with an untouched hare-lip, and the operation I did for his comfort brought the subject vividly before my mind.

